# Modeling Cut Rose Yield Over an 18‐Month Period After Compost Amendment Using Repeated Sigmoidal Gompertz Curve Fitting

**DOI:** 10.1002/pei3.70049

**Published:** 2025-04-29

**Authors:** E. A. de Nijs, A. Tietema, R. Bol, E. E. van Loon

**Affiliations:** ^1^ Institute for Biodiversity and Ecosystem Dynamics (IBED) University of Amsterdam Amsterdam the Netherlands; ^2^ Institute of Bio‐ and Geosciences, Agrosphere (IBG‐3), Forschungszentrum Jülich GmbH, Wilhelm‐Johnen‐Straße Jülich Germany

**Keywords:** compost amendment, cut roses, Gompertz curve, growth modeling, yield dynamics

## Abstract

Understanding the growth, development, and production patterns of perennial crops is crucial for optimizing agricultural practices and enhancing crop productivity. Growth models are valuable tools in this regard, offering insights into how crops respond to different experimental treatments. This study evaluates the suitability of repeated Gompertz growth curves for assessing the impact of compost amendment on the yield of cut roses over an 18‐month period. Yield data was collected from an experiment testing the effects of four different compost treatments on cut roses, with daily records of the number of stems harvested per replicate plot. Comparison of Generalized Additive Mixed Models with repeated Gompertz growth curves showed that the Gompertz model effectively captured yield dynamics in individual flushes with minimal compromise in model accuracy. As the crop matured, asymptote parameter estimates increased, while growth rate parameter estimates decreased, reflecting a stabilization of growth patterns. Compost amendment significantly enhanced early‐stage yield, with treatments receiving full fertigation consistently outperforming the control during the first year. As the crop matured, differences in yields among treatments diminished, indicating that the benefits of compost amendment are most pronounced during the initial growth phase within the 18‐month timeframe. The substantial increase in yield after compost amendment highlights its potential for sustainable management practices, guiding the sector in optimizing compost usage to enhance yield while supporting environmental sustainability. To understand the dynamic effects of different management practices (in this case different compost treatments) on rose yield across flowering flushes, the repeated growth curve provides an adequate framework.

## 1 Introduction

Optimizing crop yield is an important objective of the horticulture sector, particularly in regions where resources are limited, and sustainability is increasingly prioritized. In Kenya, where over 3000 ha (7400 acres) are dedicated to rose cultivation, crop modeling offers a valuable approach to better understand growth and yield dynamics. In addition, the use of crop models can improve production and minimize environmental impact. As the market shifts toward circular and regenerative agriculture, the use of organic amendments such as compost is gaining interest for its potential to supply nutrients and improve soil quality (Atoloye et al. 2022; Lan et al. 2022). Our study addresses these challenges by using statistical modeling to elucidate yield patterns and assess the potential of compost amendments to enhance rose production. The final aim is to provide a pathway to more sustainable and resilient agricultural practices.

Roses are a perennial crop typically cultivated in commercial settings for 6–10 years. Most of the roses in Kenya are grown in soil within polythene greenhouses (Lan et al. 2022). After the first flowering, roses are in continuous production, producing successive flowering flushes. Following the harvest of a stem, depending on the variety, one or two new stems typically reach harvest maturity in approximately 50 days. As the crop matures from a seedling to a fully mature plant, a process that takes about a year, the initially distinct flowering flushes become more uniform due to variability among individual plants. In mature crops, the length and timing of these flushes can vary based on climatic conditions and management practices.

Given the continuous production cycle and high nutrient demands of roses, maintaining soil quality is crucial for sustaining yield and quality over the years (Case et al. 2017; Fornes et al. 2024). Compost amendment to agricultural soils has been shown to improve soil quality (Bernal et al. 2017; Lim et al. 2016; Villa et al. 2021), supply and mobilize nutrients (Bergstrand 2022; Cáceres et al. 2018), and increase yield and harvest quality across various production systems (Bouhia et al. 2023; Cucci et al. 2020; Idrovo‐Novillo et al. 2019). However, the effects of compost amendments in rose cultivation on yield dynamics in modern production systems have never been reported in the scientific literature, although several studies have examined the impact of compost on crop yield in different contexts (see Gong et al. 2018; Idrovo‐Novillo et al. 2019; Liu et al. 2021; Massa et al. 2018; Ombita et al. 2024; Sossa et al. 2024). Yet, understanding these effects of different compost amendment treatments directly on yields provides valuable insights into how compost can enhance both production and sustainability in the industry.

Evaluating growth dynamics presents significant challenges, especially in perennial crops where there is a seasonal cycle but also an ontogenetic effect and possibly a carryover effect over the seasons. Statistical modeling is a powerful approach to analyze yield dynamics and thereby gain insight into the underlying patterns (Di Crescenzo et al. 2021). The methods in this study allow for the study of both ontogeny and a carryover effect by studying the progression of growth‐curve coefficients over the seasons. Sigmoidal models, such as the Gompertz curve, which describes growth as an initial slow phase followed by rapid acceleration and eventual tapering off toward a maximum limit, have proven effective in characterizing non‐linear growth (Di Paola et al. 2016). However, these models capture the distinct phases of growth, making them less suited to the repeated growth cycles seen in roses. For these more complex growth dynamics, multi‐segmented sigmoidal curves, as described by Di Crescenzo et al. (2021), provide a more suitable approach. Another, more flexible option in modeling non‐linear growth, is Generalized Additive Mixed Models (GAMM), which allow for the smoothing of complex relationships across growth phases, thereby revealing broader treatment effects (Marcillo et al. 2021).

Sigmoid growth models have been applied successfully to various crops with unique growth dynamics. Mello et al. (2023) used single sigmoid models to describe the straightforward growth pattern of sunflowers, while Fang et al. (2022) demonstrated the effectiveness of sigmoid models in simulating greenhouse tomato growth over a single season. Ukalska and Jastrzȩbowski (2019) highlighted the strength of the Gompertz model in capturing early acceleration and plateau phases in oak epicotyl emergence. Although the use of repeated sigmoid curves is less common, Fernandes et al. (2017) employed double sigmoid models to capture the two distinct growth phases of coffee berries, showing the model's capability to handle complex, multi‐phase growth patterns. While these studies often focused on identifying the best‐fitting models rather than inferring growth characteristics or comparing treatments, the application of repeated Gompertz curves in rose cultivation can offer valuable insights into how compost amendments influence yield dynamics as the crop matures.

In this study, we used a high‐resolution dataset from an 18‐month on‐farm experiment which investigated the effects of compost amendment on the yield and quality of cut roses (de Nijs et al. 2024b). This study is among the first to apply repeated sigmoidal Gompertz curve fitting to analyze the growth dynamics of cut roses as a perennial crop, under various treatments. The insights will support the practical implementation of composting in horticulture. Particularly, insight into the variations of successive flowering flushes during the growth phase will help growers in optimizing compost usage to enhance yield and contribute to sustainable resource management.

This study aimed to evaluate the impact of compost amendments on the yield of cut roses over an 18‐month period. To do so, we first developed a suitable model to describe the yield of individual flowering flushes using repeated sigmoidal Gompertz curves. Next, the model was applied to the experimental data to provide estimates of growth coefficients for different treatments across the flowering flushes. Finally, these growth coefficients were analyzed and discussed.

## 2 Materials and Methods

### 2.1 Data Collection

Yield data of cut roses was used from de Nijs et al. (2024a, 2024b) as part of a large‐scale on‐farm experiment examining the impact of compost amendment on the yield and quality of cut rose cultivation. The field experiment was conducted in a commercial greenhouse of Bilashaka Flowers, located in Naivasha, Kenya, between August 2022 and February 2024. The region experiences an average annual temperature of 18.1°C and receives an annual rainfall of 1100 mm. Cut roses, cultivated primarily for export to Europe, are grown in polythene greenhouses in soil.

The experiment consisted of five treatments, each with three replicates.

– *R*—received rose‐waste compost
– T—received rose‐waste + tomato‐waste compost
– C—received rose‐waste + mature compost
– *R* 50%—received rose waste compost but with halved fertigation dosage
– Control—did not receive compost

**FIGURE 1:**
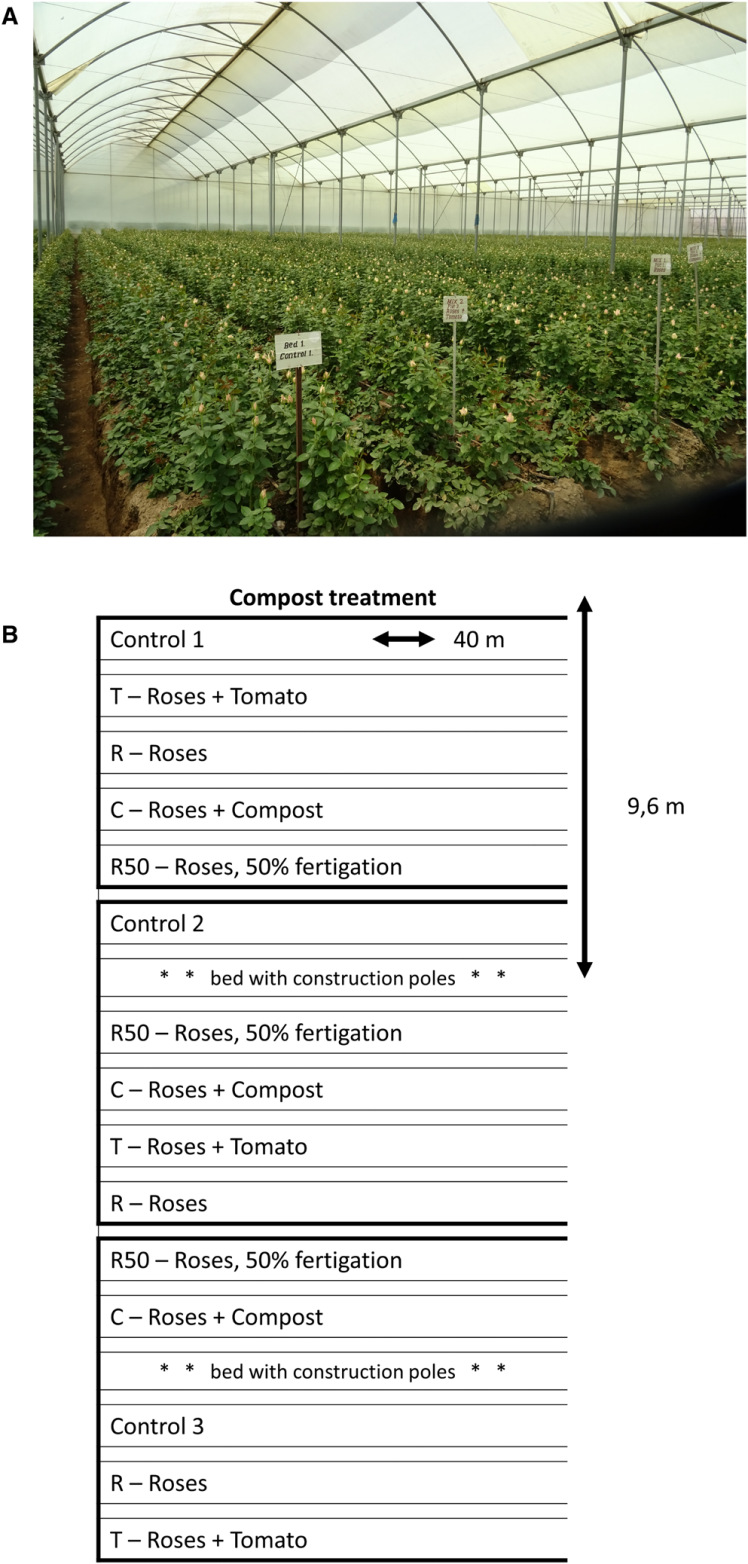
(A) Set‐up of the field experiment in the greenhouse with approximately 500 plants per bed. (B) Schematic representation, each treatment is repeated three times in three blocks, resulting in a total of 15 beds.

Compost was amended prior to planting the rose seedlings *Rosa* sp. cv. Royale Athena at a rate of 27 kg m^−2^ and incorporated to a depth of 60 cm. Approximately 500 rose plants were planted per elevated bed, with each bed measuring 44 m in length and 90 cm in width. Each bed served as a replicate, resulting in a total of 15 experimental beds. The treatments were allocated according to a randomized block design, with each block consisting of five adjacent beds, each receiving a different treatment. This block arrangement was repeated three times consecutively within the greenhouse (Figure 1A,B), and the block effect was accounted for in both modeling approaches. From the first harvest onwards, daily yield, measured as the number of stems harvested per experimental bed, was monitored for up to 18 months after the start of the experiment. Cumulative yield data per experimental bed were used for analyses to better assess differences between treatments over several flowering flushes. Further details on the experimental set‐up can be found in de Nijs et al. (2024a, 2024b). All data processing and modeling was done using R (R Core Team 2021). The dataset and the scripts are available online (de Nijs et al. 2024a).

### 2.2 Identification of Flowering Flushes

The first roses were harvested 91 days after planting the seedlings, indicating the start of the first flowering flush. It takes, depending on species and specific conditions, between 50 and 55 days for an individual new shoot to reach the right cut stage for harvest. While pronounced production flushes occurred initially, with periods of no harvest in between, variations among individual plants mitigated this pattern over time. The different flowering flushes were identified to model the flushes separately, which might give insight into plant growth development.

To identify the start of a new growth cycle (henceforth called flowering flush), we analyzed a cumulative yield dataset based on all treatments. To capture the overall trend, we tested two approaches: a Generalized Additive Model (*k*‐value = 18, thin plate spline) and a LOESS smoother (span = 0.1). Daily differences from the smoothed yield fit were calculated, and a centered 7‐day rolling mean was derived to pinpoint local minima. To ensure that the flush detection method was robust and objective, we performed a sensitivity analysis, where we varied the smoothing parameters (*k* for GAM and *span* for LOESS) and assessed how much the estimated flush start days shifted. The Root Mean Square Error (RMSE) was calculated for each variation to determine which method provided the most stable results. Based on this analysis, we selected the GAM model with *k* = 18, as it showed the lowest sensitivity to parameter changes (see Table S1 for details). There appeared to be seven minima in total, separating eight flowering flushes. Around each minimum, a temporal range of 60 days was established visually, and subsequently, the exact day at which each local minimum occurred was established algorithmically within each range. The selected time points (129, 186, 240, 302, 363, 426, 490, and 550 days after planting, see Figure S1) represent the days of minimal yield change between two flowering flushes and are in the subsequent analysis treated as starting points of a new sigmoidal growth pattern. These time points are henceforth denoted by *t*_*k*_ where *k* is the *k*‐th flowering flush. For each of these starting points, *t*_*k*_ also the cumulative yields (*y*_*k*_) are recorded. In flush 3, an unintended harvest pause introduced an artifact in the data (see Figure 3A). As a result, both the days with no harvest (days 201–213) and the subsequent catch‐up harvest period (days 214–220) were assigned a low weight (0.01), allowing the models to be fitted primarily on the beginning and end of flush 3. This approach allowed us to distinguish between flowering flushes and will help to better understand the temporal patterns of cut rose production.

### 2.3 Model Fitting

#### 2.3.1 Establishing a Benchmark

Generalized Additive Models (GAMs) are often used to model non‐linear relationships between variables (Paine et al. 2012). GAMs are semi‐parametric models which combine splines to find an optimal match to observed (nonlinear) patterns in the data. To model the cumulative number of stems harvested per treatment, we used Generalized Additive Mixed Models (GAMMs) with the default thin plate spline as base function (‘mgcv’ package). GAMMs were fitted separately for each flush similar to the Gompertz growth curve fitting (see 2.3.2). Treatment was included as a fixed effect, while individual bed (repeated measurements) and block (experimental design) were specified as random effects. A normal error distribution was used for the model (in spite of the discrete nature of the response variable), which was tested after fitting the model. The smoothing parameter (k‐value) was selected by fitting a series of GAMMs with varying k‐values for all treatment and flush combination and choosing the model with the lowest Akaike Information Criterion (AIC). A common k‐value of 5 was chosen, as further reductions in AIC were minimal (less than 2 units), achieving a balance between model complexity and fit. Model fit was evaluated by calculating Efron's *R* ^2^ values to check how accurately the models represented the observed trends in cumulative stem counts over time (Efron 1978).

#### 2.3.2 Gompertz Growth Curve Fitting

The Gompertz growth model, a sigmoidal function frequently used to describe biological growth dynamics (Di Paola et al. 2016), was chosen to analyze yield change across treatments and harvest flushes. The Gompertz growth curve is defined by the equation:

(1) $$\hat{y}=A*e^{\left(-e^{\left(B-C*t\right)}\right)}$$

where $\hat{y}$ is the estimated amount of cut roses, *A* is the upper asymptote (e.g., maximum cumulative harvest), B the growth rate, and *C* is the horizontal shift, and *t* is time in days. In this study, parameters A, B and C were fitted to the data (cumulative stem count over time since planting) as functions of treatment and experimental block. To enable fitting the model separately for each flush the variables (yield and time) were transformed to sigmoidal forms starting from zero *y* and zero *t* (the start of each flowering flush).

(2) $$y^{\prime }=A_{i}*e^{\left(-e^{\left(B_{i}-C*t^{\prime }\right)}\right)}$$

with

$$y^{\prime }=\hat{y}-y_{k}$$

$$t^{\prime }=t-t_{k}$$

In these equations the subscript *k* denotes the k‐th flush: *t*_*k*_ is the number of days after planting where the k‐th flowering flush starts and *y*_*k*_ is the cumulative yield at the start of the k‐th flowering flush. The subscript *i* denotes the individual bed (15 beds grouped over 5 treatments, including the control—see Figure 1B).

The parameters in [2] were fitted (per flush *k*) as functions of treatment as a fixed effect and Block (random effect). The individual beds were also included as random effects to account for the repeated measurement structure; note that parameter C was not varied per flower bed but kept constant. The error distributions of these parameters were assumed to be normally distributed. The assumed error distribution was checked after fitting the model.

To fit the Gompertz model, we used the ‘saemix’ package, which applies the Stochastic Approximation Expectation Maximization (SAEM) algorithm (Boedeker 2021; Comets et al. 2014). This approach allowed for the incorporation of both fixed and random effects, accounting for treatment effects, repeated measurements within individual beds, and block effect while ensuring robust parameter estimation.

The Gompertz model was fitted separately for each flush, resulting in eight distinct models, each capturing the growth dynamics within each flush period. To accurately fit the non‐linear Gompertz growth model, initial start values for each parameter were necessary to ensure effective convergence of the optimization algorithm. In this study, the starting values were set as follows: A (asymptote) at 1000, B (growth rate) at 2, and C (horizontal shift) at 0.1. These values were chosen based on preliminary data analysis to reflect realistic growth dynamics. As mentioned before (see Equation 2), parameters A and B were fitted as a function of Treatment, Block, and individual bed. However, we present one set of parameter fits per treatment for each of the eight flushes by averaging over the random factors (block and individual flower bed; see Table S2). This approach provided insights into how different treatments influenced yield patterns per flush and thereby crop maturity. The models were assessed using Efron's *R* ^2^ values, ensuring robust fitting and evaluation of growth trends across the treatments (Efron 1978).

## 3 Results

### 3.1 Data Exploration

**FIGURE 2:**
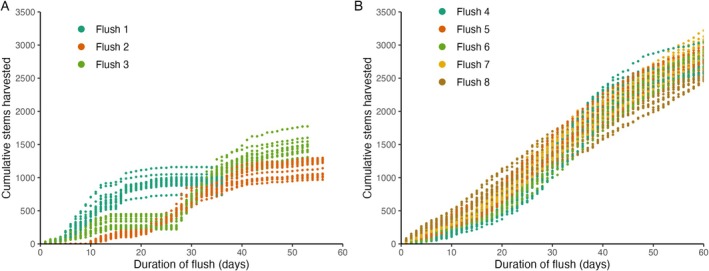
Original harvest of all treatments and replicates per flowering flush. (A) flush 1 to 3, (B) flush 4 to 8.

Cumulative yield data was split into previously defined flowering flushes to assess the impact of treatment on growth and yield dynamics in more detail as the crop matured. The growth curves of the first three flushes are quite distinct from those of flushes 4 to 8, particularly in terms of the maximum yield reached (Figure 2A,B). The first flush, which completed in 32 days, was considerably shorter than the later flushes (Figure 2A). This shorter duration may be an artifact of the chosen starting point, which was set at the first day of harvest, while the division between subsequent flushes was based on local minima in daily yield differences. Flush 3 showed a two‐week period of no yield in the middle of the flush due to a harvesting pause.

Maximum yield increased from approximately 1400 stems per flowering bed in the third flush to a consistent 2500 to 3000 stems in all subsequent flushes (Figure 2A,B), indicating that the crop was reaching production maturity. While the cumulative yield from flush 4 onwards remained relatively stable, slight variations in the rate of stem harvesting between flushes were observed. Later flushes showed a more steady and continuous increase in yield over their duration, reflecting a more consistent harvesting pattern as the crop matured.

### 3.2 GAMM to Describe Yield Patterns

**FIGURE 3:**
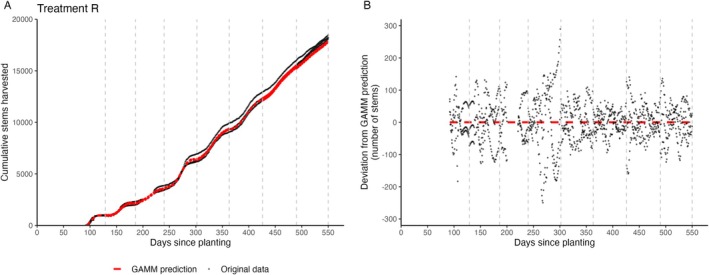
Original data points from the three replicates of treatment R (black dots) compared to the predicted yield from the fitted GAMMs (red dashed line). Vertical gray lines indicate the predetermined flowering flushes. (A) Cumulative number of stems harvested, (B) Deviation between GAMMs prediction and original data points of the three replicates.

Generilized Additive Mixed Models (GAMMs) were fitted to the cumulative stem yield data collected over the 18‐month duration of the experiment, per individual flowering flush. These models were used to evaluate and compare the overall performance across five different compost treatments, with data from three replicates per treatment to account for variability within each treatment group (Figure 3A,B).

**FIGURE 4:**
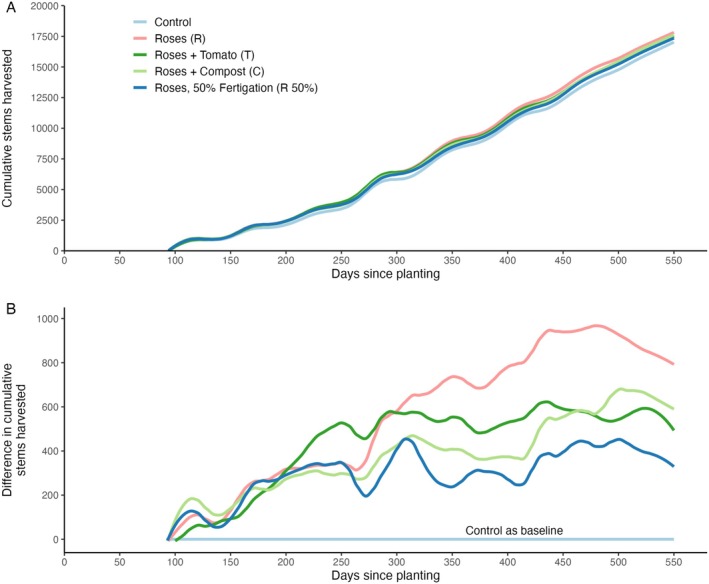
(A) Predicted cumulative stems harvested over time for five different treatments during the 18‐month experiment, based on GAMMs (*n* = 3 per treatment). (B) Differences in cumulative harvest of the four treatments compared to the control treatment.

The GAMMs effectively capture overall yield trends, as indicated with *R* ^2^ values exceeding 0.96 for the first three flushes, and 0.98 from flush 4 onwards. These high values are in part an artifact of the cumulative representation of this data: in this way, the majority of the variance in the response variable can be explained by the model (even a straight line would lead to moderately high *R* ^2^ values on a sigmoidal curve). As the differences between the replicates show in Figure 3B, a considerable amount of unexplained (and unresolvable) variability remains. Comparing overall performance between the different compost treatments showed increased cumulative yield for the three compost treatments (R, T & C) over the full 18‐month period (Figure 4A,B). Approximately 300 days after planting, the yield increase of the compost begins to stabilize (Figure 4B). Up until this point, the halved fertigation treatment (R 50%) produced yields comparable to those of the compost with full fertigation. However, after 300 days, yields for the R 50% treatment began to decline and stabilized at levels similar to the control treatment (Figure 4B).

### 3.3 Gompertz Growth Curve to Describe Yield Patterns

#### 3.3.1 Gompertz Curve Model Fits

Gompertz curve models were fitted to the data for each predetermined flowering flush, with parameter estimates for A (asymptote) and B (growth rate) varying by treatment (5).

**FIGURE 5:**
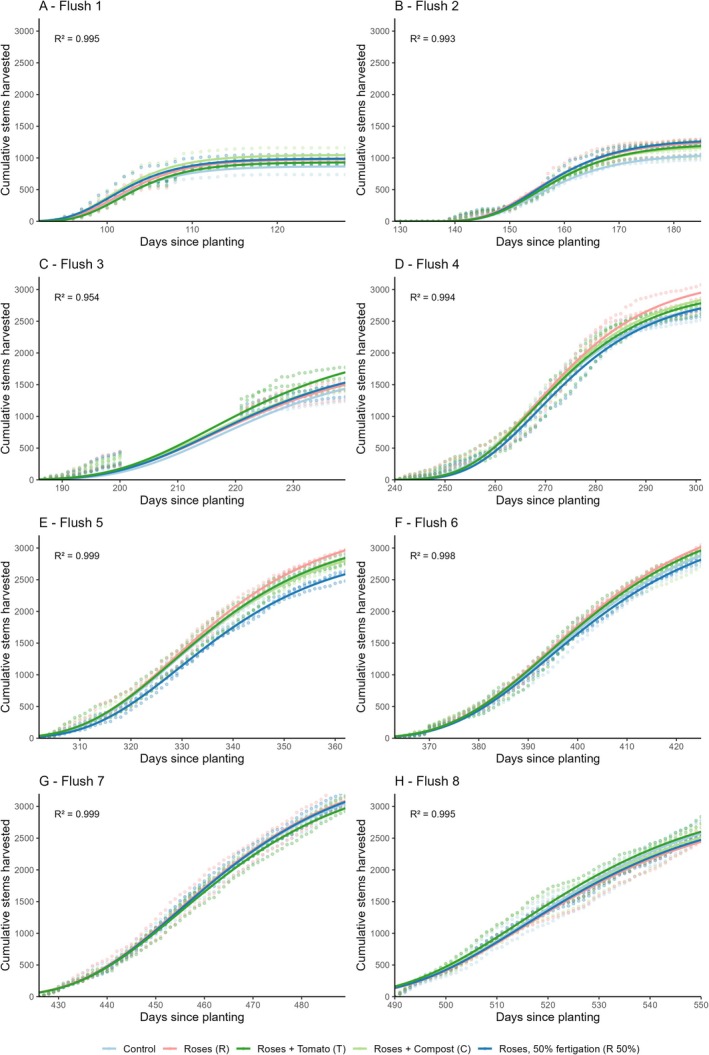
Cumulative stems harvested per flowering flush for five different treatments, shown from A—Flush 1 to H—Flush 8. The solid lines are the fitted Gompertz growth curves; dots represent the original data points (*n* = 3 per treatment). *R* ^2^ values are presented in each graph.

The models generally fit the early flushes (1–3) reasonably well, particularly during the initial growth and rapid increase phases (Figure 5A–C). However, there are some deviations visible where the original data points show variability that the models did not fully capture. This was particularly pronounced in flush 3, where a two‐week harvest stop (which was unplanned and deviated from standard operational practices) resulted in an apparent two‐step growth pattern. Therefore, we chose to omit the data of the days with no harvest (days 201–213) and the subsequent catch‐up harvest period (days 214–220) in the curve fitting to avoid a mismatch with the sigmoidal model. This resulted in a more accurate model to describe the early and late growth pattern in this flush with an *R* ^2^ value of 0.954, well in line with that of flush 1 (0.995) and flush 2 (0.993). This reflects the model's strength to capture growth patterns during this period.

Flushes 4 to 6 show similar behavior and fit (Figure 5D–F). As the crop matures, the model fits become smoother and more closely aligned with the original data points, suggesting that the Gompertz growth curve performs better as growth dynamics become more consistent. This is reflected by the increasing *R* ^2^ values, from 0.994 in flush 4 to 0.998 in flush 6.

In flush 7 and 8, the yield increase during each flush is more gradual, as noted previously in 3.2.1 (Figure 5G,H). The transition to this more gradual yield increase is slightly less accurately captured by the Gompertz models, as the observed data slightly exceed the predicted values toward the end of both flushes. Despite this, the model fit remains strong, with *R* ^2^ values of 0.999 and 0.995 for flush 7 and 8, respectively.

A comparison of the GAMMs fit and the Gompertz curve fit for each flowering flush showed a relatively similar pattern, with the largest variations between the two modeling approaches occurring at the beginning and end of a flush (Figure S2).

#### 3.3.2 Rose Yield Dynamics

**FIGURE 6:**
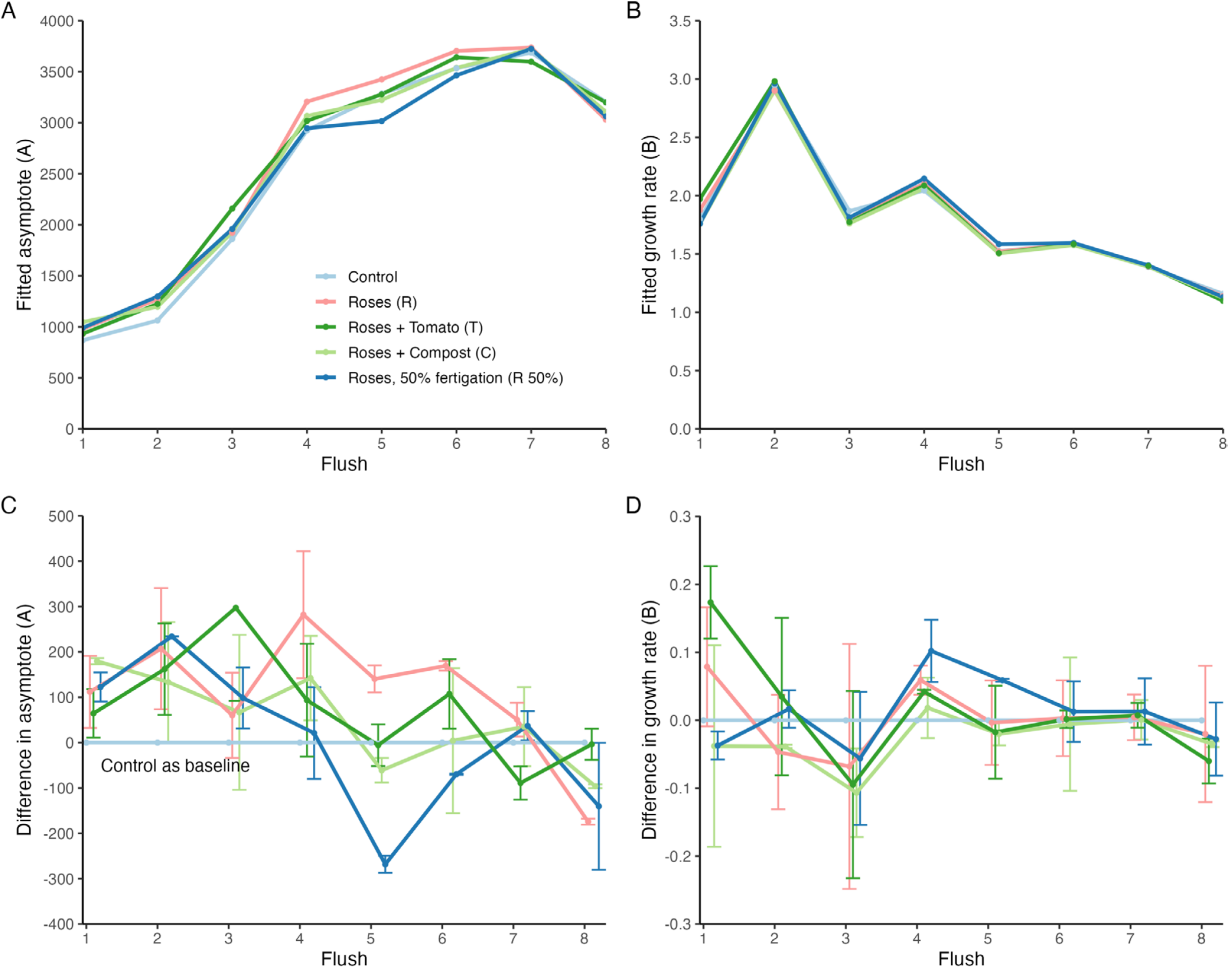
Fitted parameter values from the Gompertz growth curves per treatment over the eight flowering flushes for (A) the asymptote A and (B) growth rate parameter B, standard error is not presented for readability. Panel (C) and (D) show the differences in parameter estimates between the four treatments and the control treatment, with bars indicating the 95% confidence intervals.

Rose yield dynamics across eight flowering flushes, as measured by cumulative stems harvested, were expressed by the asymptote (A) and growth rate (B) of the growth curves. The progression of these parameters over the flowering flushes is shown in Figure 6A and shows distinct performance patterns among the tested compost treatments. The horizontal shift parameter (C) was fitted individually for each flush but was not subjected to further analysis (Table S2).

The control treatment consistently showed the lowest yield in the initial flushes, as reflected in both cumulative yield and asymptote A values (Figure 6A). However, these differences diminished over time as the yield of the other treatments became more comparable in the later flushes (Figure 6C). This pattern indicated that while compost amendments significantly enhance early‐stage yield, their effects disappeared as the crop matures. Treatments R, T, and C, all receiving compost and full fertigation, consistently outperformed the control, particularly during the first six flushes (Figure 5 & Figure 6A,C). The differences between the treatments and the control with respect to the model parameters A and B were not significantly different for all flushes; however, they were especially significant for flushes 4 to 6 (Figure 5C,D). While the confidence intervals for the asymptote (parameter A)  remained rather similar over time (indicating constant differences among the repetitions), those for the growth rate (parameter B) narrow substantially during the later flushes. Hence, a higher precision of the growth rate was obtained as the crop stabilized in its production (Figure 6C,D). The higher initial growth rates (B) in these treatments supported the effectiveness of compost amendment in promoting rapid early growth, with yields peaking between flush 4 and 6 (Figure 6B,D). By the last two flushes, the differences in yield potential (A) and growth rate (B) between these treatments and the control diminished, indicating that the crop's maturation eventually leveled out performance differences within this timeframe. The R 50% treatment, which received compost but only half the standard fertilizer dosage, initially performed comparably to the fully fertigated treatments, as shown by similar cumulative yields and asymptote values up to flush 4. However, from flush 5 onward, its yield began to lag, leveling off at yield levels similar to those of the control. This indicated that while compost amendment might sustain growth in the short term with halved fertigation, it may not be sufficient to maintain optimal yield over the entire 18‐month period.

The asymptote parameter A increased from approximately 1000 to 3500 stems per flowering bed per flush as the rose crop matured (Figure 6A). The slight decline observed in the last two flushes may be attributed to climatic conditions (colder periods) or management practices. The growth rate parameter B was relatively low across all treatments in the first flush, likely due to the chosen starting point, which was the first day of harvest, making flush 1 shorter. Over time, the growth rate decreased across all treatments, indicating crop maturation, where variations among individual plants smoothed the initially pronounced production flushes (Figure 6B,D).

## 4 Discussion

### 4.1 Suitability of Gompertz Model for Repeated Growth Curves

Modeling biological yield in perennial crops is especially challenging due to overlapping growth phases and variable flushes. In this study, the suitability of the sigmoidal Gompertz growth curve for assessing the effect of compost amendment on rose yield across multiple flowering flushes was evaluated by comparing repeated Gompertz curves with a Generalized Additive Mixed Model (GAMM). A GAMM is very flexible in fitting non‐linear shapes, and we use it as a benchmark against which the predictive performance (here through the Effron *R* ^2^) of the Gompertz growth curve is compared. One advantage of modeling consecutive flowering flushes separately with a parametric model is the opportunity to gain insights into yield patterns as the crop matures. This is particularly relevant for rose crops that are in production for 6 to 10 years. During this period, growth dynamics change considerably from seedlings to mature plants and are further influenced by local climatic conditions and maintenance practices (Figure 2, flush 1–3 vs. 4–8). The Gompertz model's repeated sigmoidal curve approach proved especially useful for analyzing differences in growth rates and maximum yields across flowering flushes as the crop matured.

While GAMMs are very effective in capturing broad treatment effects with very high *R* ^2^ values and evaluating variability between treatments, they do not provide parameters that can be interpreted biologically (like growth rate and maximum yield). In this study, the Gompertz model effectively captured the stem yield pattern of cut roses, as supported by generally consistent fits and *R* ^2^ values ranging from 0.954 to 0.999 (Figure 5). However, the model did not fully capture the asymptotic behavior at the end of the flushes, particularly in the early flush where there is a harvest pause between flushes. For example, in flush 4 (Figure 5D), the fitted curve slightly overestimated the actual cumulative yields. This discrepancy became less pronounced in subsequent flushes where model predictions and observed yields aligned well (Figure E, F). Yet, as the crop reached full maturity in flushes 7 and 8, cumulative yields began to show a less pronounced asymptotic behavior, leading to slight underestimations by the sigmoidal model (Figure 4G,H). As the crop matured beyond the study's 18‐month period, yield patterns were likely to be increasingly influenced by climatic variability and management practices, potentially requiring adjustments or supplementary models for accurate long‐term yield predictions.

No systematic trends in model bias and accuracy were observed within as well as between flushes, in spite of yield variations in the young crop and a less pronounced sigmoidal curve pattern in the fully matured crop. The Gompertz model provided a reliable and accurate representation of overall yield dynamics across multiple flowering flushes. While the application of repeated sigmoidal curves to flower yield has not been extensively studied, research on other crops highlights their potential for capturing growth patterns. For example, the Gompertz model effectively described sunflower growth within a single season, outperforming the logistic model (Mello et al. 2023), likely due to its non‐symmetrical inflection point. In pecan nuts, model preference varied by developmental stage (Panta et al. 2023). For perennial crops like coffee, growth followed a repeated sigmoidal pattern, with both logistic and Gompertz models performing well (Fernandes et al. 2017). Although the logistic model had a slight advantage in early development stages, possibly due to the sampling intervals, similarly, greenhouse‐grown tomatoes over two crop seasons were better fitted by the logistic model based on five biologically relevant critical points (Fang et al. 2022). Collectively, these studies support the broader applicability of sigmoidal models as tools for understanding growth and yield patterns.

Direct comparisons between the Gompertz growth curve and the more flexible GAMM model in our study showed that both approaches produced very similar fits for each flowering flush (Figure S2). Nevertheless, this study showed that GAMMs were slightly better at capturing the less pronounced asymptotic behavior (Figure S2G,H), whereas the Gompertz growth curve was more suitable for modeling rose harvest at the end of the flushes. The Gompertz curve offered an important advantage: it allowed for direct comparison of treatment effects across different flowering flushes, which is an important advantage in understanding and optimizing rose production. Using GAMMs as a baseline model for sigmoidal patterns to evaluate the performance of specific sigmoidal growth curves is, to our knowledge, a novel approach. We found a study where the growth of different types of woody biomass was modeled using various functions, including the Gompertz curve and GAM, but direct comparisons across all growth stages have been limited (Jevšenak et al. 2022).

The Gompertz curve is well‐suited for monophasic growth and can be combined in an additive fashion to capture more complex patterns like bimodal growth (Fernandes et al. 2017). However, the number of parameters in this model type adds up in multiphasic situations (typically for more than 3 phases) so that fitting all of the parameters in a single model is not feasible. We addressed this problem by separating the data into distinct growth phases (flushes) and subsequently fitting the monophasic growth models sequentially per flush. This allows the Gompertz function (and potentially alternative monophasic growth models as well) to describe growth within each period separately. Using separate models per flush allows us to compare parameter estimates across flushes (as shown in Figure 6), and also to make cumulative predictions (over multiple flushes). However, confidence intervals (or other uncertainty estimates) for cumulative fluxes cannot be based on the cumulative models in a straightforward way. To calculate uncertainties for cumulative predictions, one would have to assume independence in parameter uncertainties among the models (or know their covariance structure). Judging from Figure 6, we observe some correlation in parameter uncertainty ranges between consecutive flushes and would therefore refrain from making this assumption. In future work, it would be valuable if a method were developed to model the error propagation across subsequent growth phases so that cumulative predictions would be accompanied by uncertainty estimates.

In this study we have fitted a semi‐parametric model (GAMM) in addition to the growth curve model so that we could put the goodness‐of‐fit statistic as well as model residuals for our model in context. In this case we found that the growth curve performed surprisingly well—prior to the analysis we expected a larger gap between the goodness of fit by the GAMM and Gompertz models. It emphasizes that the Gompertz model provides reasonable estimates. Nonetheless, some structural deviations between model predictions and observed growth remain. These do however differ qualitatively among the different flushes and we therefore do not expect that a different growth model would resolve this.

Nonetheless, it would be valuable to evaluate alternative growth curves that have been shown to perform well for roses (Cao et al. 2019; Ukalska and Jastrzȩbowski 2019) in a follow‐up study. However, these alternative models are usually more complex. While additional parameters tend to capture greater variability in the data, they also make model interpretation more challenging. This balance between accuracy and interpretability is crucial when selecting a growth model for practical applications in horticulture.

### 4.2 Rose Yield Dynamics After Compost Amendment

Analyzing rose yield dynamics across eight flowering flushes using Gompertz growth curves provided a detailed analysis of how compost amendments influence rose production as the crop matures. The asymptote (A) and growth rate (B) parameters derived from these models show distinct performance patterns among the tested compost treatments, revealing the benefits and limitations of compost use in rose cultivation.

A clear ontogenetic development of the maturing crop is visible up until flush 4, with increasing asymptotic values (Figure 6A). However, an apparent decrease in maximum cumulative yield (A) was observed in flush 8 across all treatments (Figure 6A), which occurred from late December 2023 to early February 2024. The uniform decline across all treatments suggested a weather‐related impact, as no significant management decisions were taken during this period. An inquiry learned that heavy rainfall during this period led to an outbreak of downy mildew, a fungal disease that causes leaf abscission, leading to a substantial growth loss. The variations in growth rate between later flushes (Figure 5B) might indicate carry‐over effects, where significant growth in one flush leads to reduced growth in the subsequent flush. However, no existing literature supports a substantial role of this phenomenon in rose yield. The impact of carry‐over effects is likely minimal, as the number of shoots generally increases with each flush. Through crop management practices, such as regulating shoot number via strategic pruning, growers maintain 6 to 8 active shoots per plant, ensuring consistent productivity.

Parameter fits clearly indicated how compost amendments significantly enhanced early‐stage yield, as reflected by the increased asymptotic values (A) in the first four to six flushes (Figure 5A,C). Treatments R, T, and C, which received compost and full fertigation, consistently outperformed the control treatment, particularly during flushes 4 to 6, where growth rates (B) were slightly elevated compared to the control (Figure 5B,D). Although growth rates during these flushes were not significantly higher than for the control, the predicted maximum yields were higher, indicating that compost amendments effectively promote rapid early growth, leading to higher yields during critical growth phases while maintaining soil health. As the crop matured, the benefits of compost amendment on yield appeared to diminish. By the last two flushes, the differences in yield potential (A) and growth rate (B) between the compost treatments and the control became less pronounced. The narrowing of confidence intervals in the later flushes further supported this observation, indicating a convergence in yield and growth rate across all treatments as the crop's production stabilized. This trend suggested that while compost amendments are particularly effective in enhancing early‐stage yield, their impact may level off as the crop reaches maturity within the first 18 months after planting. These findings align with previous projections from this experiment from de Nijs et al. (2024a, 2024b), where overall yield was significantly enhanced by compost amendment, but mature yield between 12 and 18 months was not. This might be caused by the early depletion of the readily available nutrients from the compost as it coincides with the reduced performance of the R 50% treatment, or the crop's reduced responsiveness to nutrient inputs at later stages of growth.

The long‐term benefits of compost amendments on yield may become more apparent in later growth stages, particularly as the advantage of improved soil functioning and slow release of nutrients becomes more pronounced over time. This aligns with findings from a meta‐analysis by Luo et al. (2018), who observed that 3 years post‐application, the impacts of organic amendments on crop yield increased. Similar yield increases were reported in studies on roses by Barbosa et al. (2019) and on millet by Meena et al. (2016), where the combined application of organic and mineral fertilizers significantly enhanced crop productivity. This synergy between organic and mineral fertilizers not only boosted yield but also reduced dependency on mineral inputs (Bernal et al. 2017). The lasting effects of compost amendment across multiple production cycles were attributed to both the nutritive and non‐nutritive benefits provided by organic matter (Reeve et al. 2012).

The R 50% treatment, which combined compost amendment with only half the standard mineral fertilizer dosage, offers additional insights into the nutrient provision role of compost. This treatment sustained yields comparable to those of the compost + fully fertigated treatments up to flush 4. However, from flush 5 onward, yields began to decline and eventually stabilized at levels similar to the control (Figure 5A). Although reducing fertigation is feasible, a 50% reduction may not sufficiently support enhanced productivity over time. This has important implications for the horticultural sector, as it indicates that reductions in mineral fertilizer use are possible when applying compost.

Substantial yield variations between replicates were observed in this study, as clearly depicted in Figure 3B. While the experiment included three repetitions per treatment, which resulted in significant effects, this number might be rather small considering the observed variability. Therefore, future experiments of this nature should consider increasing the number of replicates. This would enhance the statistical power, leading to more robust conclusions about the impact of different treatments on yield.

The consistent fit of the Gompertz model across different treatments and flushes demonstrated its effectiveness in modeling yield in rose cultivation, offering valuable insights into the crop's growth patterns. The model successfully captured the key phases of growth, peak production, and plateau, providing a comprehensive understanding of how different compost treatments influenced yield dynamics. While this model proved adequate for describing and interpreting rose yield data, other growth curves, such as the logistic equation, Richards, or ontogenetic growth models (Cao et al. 2019), could also be suitable or even more appropriate in certain cases. The framework presented in this study allows for the straightforward exploration of different growth curves to find the best fit for specific yield data.

Utilizing repeated sigmoidal curves to analyze yield dynamics provides valuable insights into how compost management practices affect rose cultivation, potentially aiding farmers in making more informed decisions. Future research should refine the model to better account for climatic variability and management practices beyond the 18‐month mark, further enhancing its applicability in predicting long‐term yield outcomes. Moreover, considering the insights provided by Di Crescenzo et al. (2021) on the application of multi‐sigmoidal models in plant dynamics, integrating such approaches may offer even more nuanced understanding and predictive capabilities for long‐term yield optimization in rose cultivation.

## 5 Conclusion

Repeated sigmoidal growth models showed to be an effective approach to model the yield dynamics of cut roses under different compost treatments over time. This study presented an analytical framework for repeated growth curve analysis by comparing the Gompertz curve, a more interpretable parametric growth curve, with the more flexible but less interpretable non‐parametric GAMM. To accurately split growth stages, in this case flowering flushes, the local minima of yield change were identified. Model fits of the separate Gompertz curves were directly compared to those of the GAMMs. The Gompertz curve fitted per flowering flush successfully captured key phases of growth and peak production across multiple flowering flushes, with *R* ^2^ values ranging from 0.954 to 0.999. Its ability to distinguish growth patterns between flushes and treatments makes it a valuable tool for optimizing compost management practices in rose cultivation. While the Gompertz model performed well, this framework allowed for the straightforward testing and comparison of other growth models, which may perform equally well or better. The insights gained from this modeling approach highlight that compost amendments significantly boosted yield during the initial months. However, these benefits diminish as the crop matures, potentially offering practical guidance for sustainable production practices in the horticultural sector.

## Conflicts of Interest

The authors declare no conflicts of interest.

## Supporting information


Data S1.

## Data Availability

The data and scripts that support the findings of this study are openly available in Zenodo at https://doi.org/10.5281/zenodo.13772891.
