# Supplementary material for: Modeling Cut Rose Yield Over an 18‐Month Period After Compost Amendment Using Repeated Sigmoidal Gompertz Curve Fitting
Source: Plant Environ Interact. 2025 Apr 29;6(3):e70049. doi: 10.1002/pei3.70049 (PMC12041442; doi:10.1002/pei3.70049)
Supplement: Supplementary file 1 — Data S1. [file PEI3-6-e70049-s001.docx]

Supplementary data

Table S1. Sensitivity analysis results for flowering flush detection using two smoothing approaches: Generalized Additive Models (GAM) and LOESS. The table presents the Root Mean Square Error (RMSE) values for variations in the GAM smoothing parameter (k) and the LOESS smoothing parameter (span), relative to the baseline model (k = 18, span = 0.10).

|  | GAM | | | | LOESS | | | |
| --- | --- | --- | --- | --- | --- | --- | --- | --- |
|  | k=17 | **k=18** | k=19 | k=20 | span=0.09 | span=0.10 | **span=0.11** | span=0.12 |
| RMSE value (compared to baseline) | 2.236 |  | 0.756 | 0.756 | 14.152 |  | 0.926 | 1.254 |
| Cutoff days | | | | | | | | |
| flush 1 | 149 | 129 | 129 | 129 | 125 | 125 | 125 | 125 |
| flush 2 | 160 | 186 | 186 | 187 | 202 | 180 | 179 | 182 |
| flush 3 | 220 | 240 | 241 | 241 | 235 | 239 | 240 | 240 |
| flush 4 | 301 | 302 | 303 | 302 | 297 | 298 | 298 | 298 |
| flush 5 | 368 | 363 | 363 | 363 | 363 | 363 | 363 | 364 |
| flush 6 | 439 | 426 | 425 | 425 | 430 | 429 | 427 | 427 |
| flush 7 | 512 | 490 | 489 | 491 | 520 | 490 | 490 | 489 |
| flush 8 | 550 | 550 | 550 | 550 | 550 | 550 | 550 | 550 |


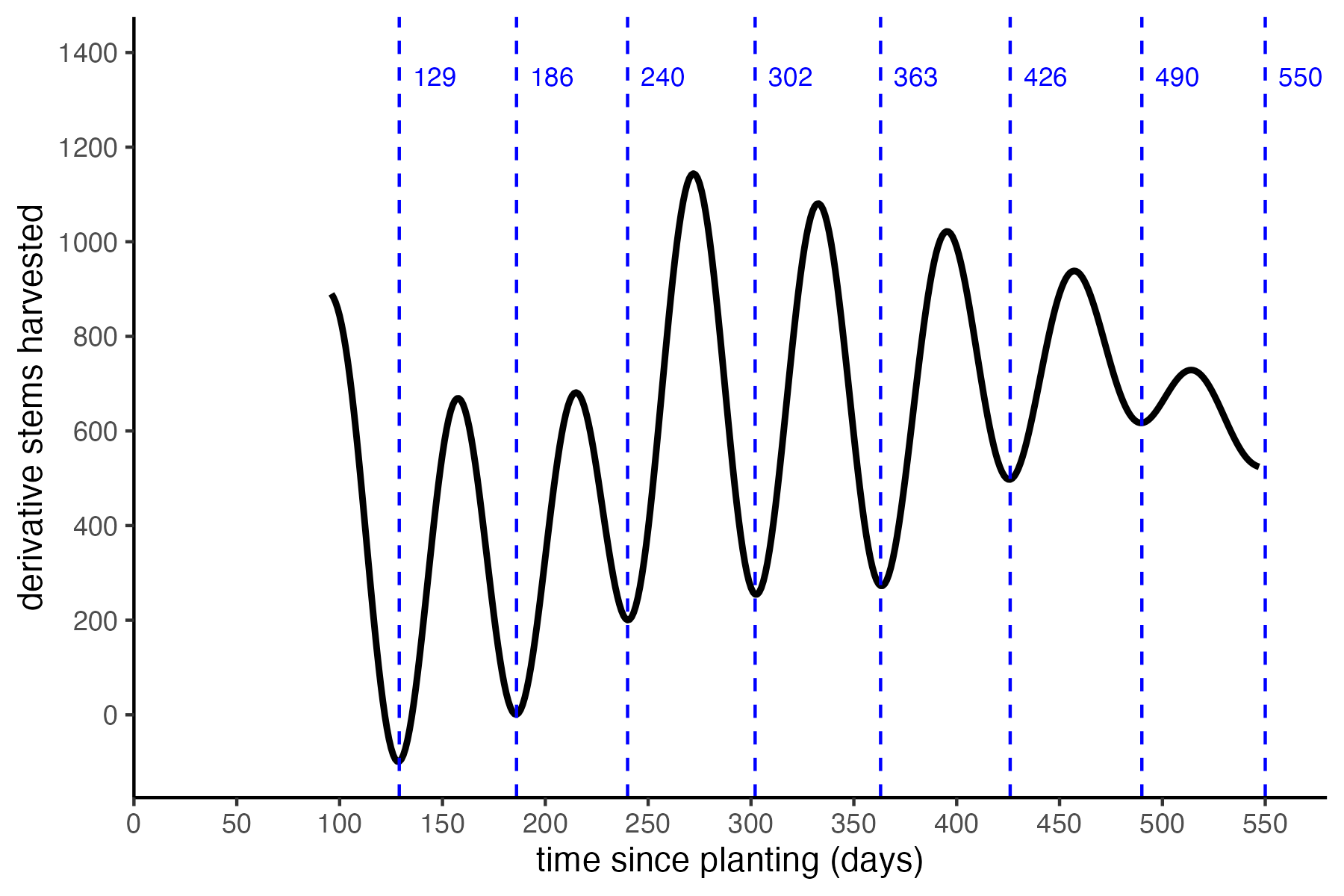


Fig S1. Smoothed derivative of cumulative yield of cut roses including all experimental beds with the identified flowering flushes indicated by the blue dashed lines.

Table S2. Gompertz curve parameter estimates A and B per treatment as modelled for each flush. Parameter C was fitted per flush. Compost treatments: control, R = 100% roses, T = 80% roses + 20% tomato, C = 90% rose waste + 10% mature rose compost, R 50% = 100% rose waste which received 50% of the fertigation.

| Flush | Treatment | Parameter A | Parameter B | Parameter C |
| --- | --- | --- | --- | --- |
| 1 | control | 868 | 1.80 | 0.22 |
| 1 | R | 980 | 1.88 |  |
| 1 | T | 932 | 1.97 |  |
| 1 | C | 1047 | 1.76 |  |
| 1 | R 50% | 990 | 1.76 |  |
| 2 | control | 1063 | 2.95 | 0.12 |
| 2 | R | 1270 | 2.90 |  |
| 2 | T | 1225 | 2.98 |  |
| 2 | C | 1196 | 2.91 |  |
| 2 | R 50% | 1297 | 2.96 |  |
| 3 | control | 1861 | 1.87 | 0.06 |
| 3 | R | 1921 | 1.80 |  |
| 3 | T | 2159 | 1.77 |  |
| 3 | C | 1928 | 1.76 |  |
| 3 | R 50% | 1960 | 1.81 |  |
| 4 | control | 2924 | 2.04 | 0.08 |
| 4 | R | 3206 | 2.10 |  |
| 4 | T | 3018 | 2.09 |  |
| 4 | C | 3067 | 2.06 |  |
| 4 | R 50% | 2946 | 2.15 |  |
| 5 | control | 3284 | 1.52 | 0.06 |
| 5 | R | 3425 | 1.52 |  |
| 5 | T | 3279 | 1.51 |  |
| 5 | C | 3223 | 1.50 |  |
| 5 | R 50% | 3016 | 1.58 |  |
| 6 | control | 3543 | 1.58 | 0.05 |
| 6 | R | 3703 | 1.58 |  |
| 6 | T | 3641 | 1.58 |  |
| 6 | C | 3538 | 1.58 |  |
| 6 | R 50% | 3464 | 1.59 |  |
| 7 | control | 3687 | 1.39 | 0.05 |
| 7 | R | 3737 | 1.39 |  |
| 7 | T | 3598 | 1.40 |  |
| 7 | C | 3722 | 1.39 |  |
| 7 | R 50% | 3724 | 1.40 |  |
| 8 | control | 3202 | 1.16 | 0.04 |
| 8 | R | 3029 | 1.14 |  |
| 8 | T | 3199 | 1.10 |  |
| 8 | C | 3107 | 1.12 |  |
| 8 | R 50% | 3063 | 1.13 |  |


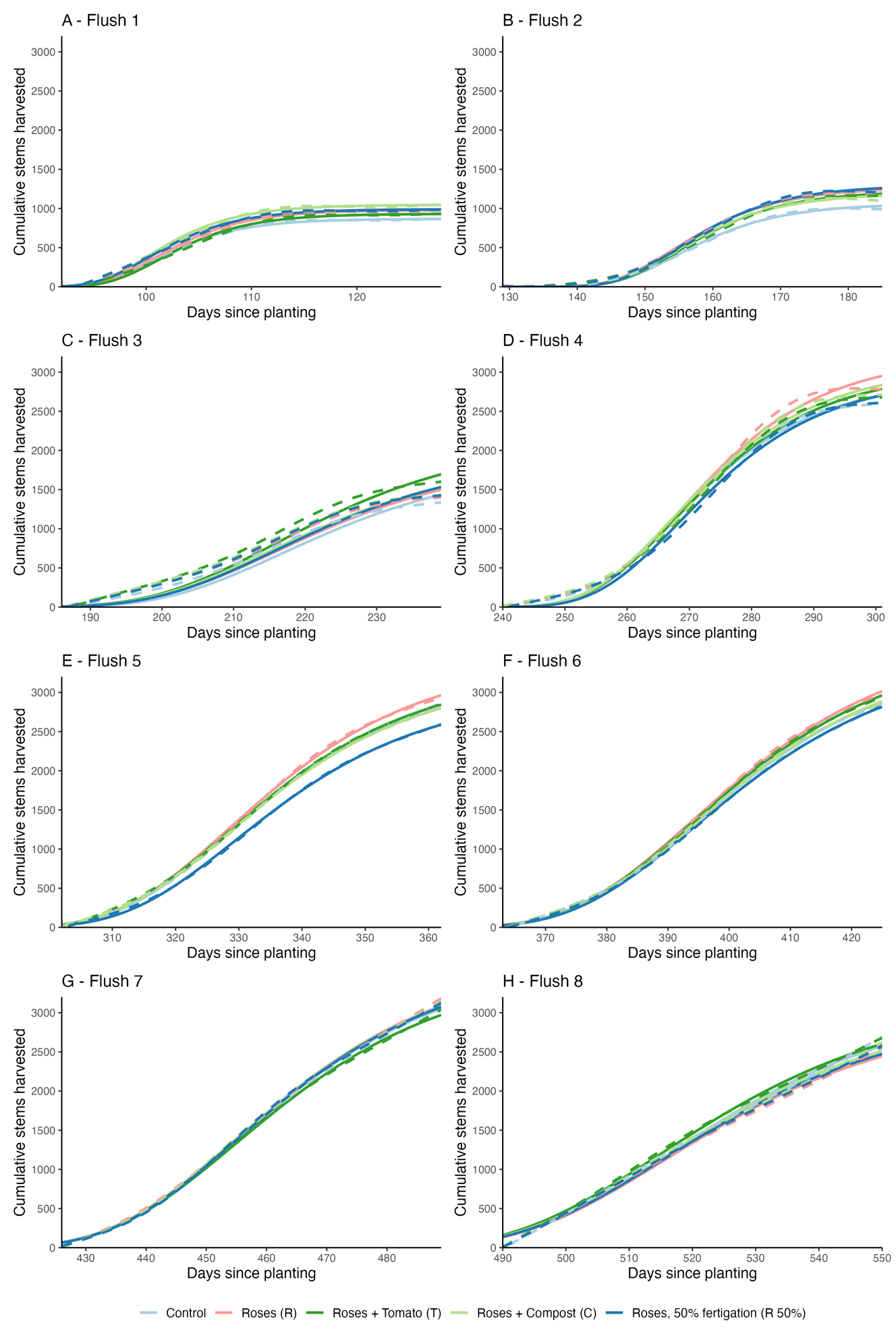


Fig S2. Modeled cumulative stems harvested per flowering flush for five different treatments, shown from A – Flush 1 to H – Flush 8. The solid lines represent the predicted Gompertz growth curve fits, dashed lines represent the predicted GAMMs fit.
